# The architectural landscape of diverse ciliary functions

**DOI:** 10.1186/2046-2530-4-S1-O13

**Published:** 2015-07-13

**Authors:** M Bettencourt-Dias, S Chandra Jana, P Machado, J Rocha, S Mendonça, S Werner

**Affiliations:** 1 Instituto Gulbenkian de Ciencia, Oeiras, Portugal

## 

Cilia are microtubule-based membrane protrusions conserved across evolution involved in cell motility, fluid flow and sensing.

The diversity in functions is generally attributed to a core conserved microtubule-based structure, the axoneme, decorated by different structures, membrane and signaling systems. Here we study four classes of cilia that represent very diverse motility and sensory functions within a single organism, the fruit fly. We uncover that the base of the cilia, the basal body and transition zone, is much more diverse than previously thought, showing large variation in number, length, ultrastructure, and connection to other cellular structures. We further demonstrate that basal body diversity is imparted by differential regulation of evolutionarily conserved core components. The tissue specific regulation of core basal body and transition zone genes suggests mechanisms that generate tissue specific phenotypes in human ciliopathic syndromes.

